# Population need for primary eye care in Rwanda: A national survey

**DOI:** 10.1371/journal.pone.0193817

**Published:** 2018-05-01

**Authors:** Tess Bright, Hannah Kuper, David Macleod, David Musendo, Peter Irunga, Jennifer L. Y. Yip

**Affiliations:** 1 International Centre for Eye Health, Faculty of Infectious Disease and Tropical Diseases, London School of Hygiene & Tropical Medicine, Keppel Street, London, United Kingdom; 2 International Centre for Evidence in Disability, Faculty of Infectious Disease and Tropical Diseases, London School of Hygiene & Tropical Medicine, Keppel Street, London, United Kingdom; 3 Department of Infectious Disease Epidemiology, Faculty of Epidemiology and Population Health, London School of Hygiene & Tropical Medicine, Keppel Street, London, United Kingdom; 4 Lifetime Consultants, Kigali, Rwanda; National Yang-Ming University Hospital, TAIWAN

## Abstract

**Background:**

Universal access to Primary Eye Care (PEC) is a key global initiative to reduce and prevent avoidable causes of visual impairment (VI). PEC can address minor eye conditions, simple forms of uncorrected refractive error (URE) and create a referral pathway for specialist eye care, thus offering a potential solution to a lack of eye health specialists in low-income countries. However, there is little information on the population need for PEC, including prevalence of URE in all ages in Sub-Saharan Africa.

**Methods:**

A national survey was conducted of people aged 7 and over in Rwanda in September-December 2016. Participants were selected through two-stage probability proportional to size sampling and compact segment sampling. VI (visual acuity<6/12) was assessed using Portable Eye Examination Kit (PEEK); URE was detected using a pinhole and presbyopia using local near vision test. We also used validated questionnaires to collect socio-demographic and minor eye symptoms information. Prevalence estimates for VI, URE and need for PEC (URE, presbyopia with good distance vision, need for referrals and minor eye conditions) were age and sex standardized to the Rwandan population. Associations between age, sex, socio-economic status and the key outcomes were examined using logistic regression.

**Results:**

4618 participants were examined and interviewed out of 5361 enumerated (86% response rate). The adjusted population prevalence of VI was 3.7% (95%CI = 3.0–4.5%), URE was 2.2% (95%CI = 1.7–2.8%) and overall need for PEC was 34.0% (95%CI = 31.8–36.4%). Women and older people were more likely to need PEC and require a referral.

**Conclusions:**

Nearly a third of the population in Rwanda has the potential to benefit from PEC, with greater need identified in older people and women. Universal access to PEC can address unmet eye health needs and public health planning needs to ensure equitable access to older people and women.

## Introduction

Vision loss is a leading cause of disability worldwide. An estimated 624 million people live with visual impairment (VI), including 19 million children. [1] Visual impairment infers a considerable economic burden to the individual, family and society through the costs of lost productivity and care.[2] Prevention of blindness has been a priority for the World Health Organisation (WHO) for nearly twenty years through its partnership programme with the International Agency for Prevention of Blindness (IAPB) in the VISION 2020: The Right to Sight Programme. The main premise behind this initiative is that up to 80% of blindness is avoidable, that is, treatable or preventable, if services are available.[3]

The most common cause of VI is uncorrected refractive error (URE), where the eye fails to focus light rays clearly onto the retina.[4] The resulting blurred vision can be corrected with glasses. An age related decline in near vision due to loss of lens elasticity (presbyopia) is also common, and correctable with glasses. Glasses can be typically provided in a community setting without the need for prescription.[4–6] Cataract is the most common cause of severe VI in Africa, and can be treated through a simple operation.[7]

Despite the simple and cost effective intervention for URE, few people who need glasses have them, with low spectacle coverage in Africa ranging from 7% in the Gambia to 54% in Botswana for distance URE.[8] Coverage of spectacles for presbyopia is even lower.[8] However, data is sparse, particularly for children and young adults in the African region.[9] Additionally, uptake of cataract surgery is also low in many sub-Saharan African countries, in part due to lack of referrals to existing services. [10] Lack of eye care services, including a severe shortage of trained eye health workers, are therefore a major barrier to the achievement of the VISION 2020 goals.[6] Efficient and accessible health services, where the minor conditions are treated in the community and more serious conditions are referred to specialist care, are important in developing health services that can maximize use of limited resources and improve eye health outcomes.

Integrated primary eye care (PEC), where general health nurses are trained to provide primary eye health services in addition to general health services, is a potential solution to address the lack of specialist eye health workers in low-income settings and scale up eye care. PEC can address most forms of URE and refer the rest for specialist treatment. Other conditions that can be addressed in PEC include common minor eye diseases–including allergic or infectious conjunctivitis and dry eyes–which can be treated with eye drops or health information.[11] Consequently, in 2013, the World Health Assembly adopted a new global action plan for the prevention of avoidable blindness, which positions universal access to PEC as the cornerstone of the VISION 2020 global initiative.[12]

PEC programmes have been attempted in several low income countries in sub-Saharan Africa including Tanzania, Rwanda and Malawi.[13] However, there is very little evidence of effectiveness. Results of a pilot studies in Zanzibar and Dar-es-Salam suggest that primary health care workers could distribute presbyopic corrections and improve staff knowledge and practices around child eye health respectively, however these effects was not sustained.[14, 15] Evidence from Chikwawa district in Malawi suggests traditional healers may play a role in improving cataract surgical uptake.[16]

This study is set in Rwanda, a low-income country in East-Central Africa with a predominantly rural population of 11.6 million.[17]. Previous attempts at introducing PEC into Rwanda had limited impact, due to the shortage of trained nurses and lack of consistency in care provided.[18] More recently, a new PEC curriculum has been developed and introduced into the national nursing training programme with work towards a national programme of sustainable PEC.[19]

Though PEC is a major global eye health initiative, there is limited evidence of its potential impact and whether it can meet the needs of the target population.[13] This study aims to examine the background need for, and potential to benefit from PEC in Rwanda.[19].

## Methods

A national survey was conducted between 26^th^ September 2016 and 31^st^ December 2016 in 10 districts of Rwanda. We based the sample size on the expected prevalence of visual impairment of 3%, as a key outcome for PEC is detection of VI for referral.[1] The other key outcomes (need for glasses, need for eye drops) were expected to be more common, and thus the sample size would be adequately powered for all outcomes. Based on the estimated prevalence of 3%, 20% precision, 90% response rate, a design effect of 1.4 and confidence levels of 95%, a sample size of 4800 was required, which would be recruited in 50 clusters of 96 people.

### Sample selection

We used a two-stage sampling process with probability proportionate to size (PPS) procedures, using 2012 census data with projections to 2016 (produced by the National Institute of Statistics Rwanda) as the sampling frame. The first stage selected 10 districts from a list of all districts and their corresponding populations derived from the census. The selected districts included three from the Western province (Nyamasheke, Karongi, Rutsiro); two from the Southern province (Kamonyi, Nyanza); two from the Northern province (Gakenke, Rulindo); two from the Eastern province (Gatsibo, Ngoma); and one from Kigali province (Kicukiru). Following this, five villages from each district were selected as clusters, again using PPS.

We selected households in each cluster using compact segment sampling. Each cluster was divided into segments each containing approximately 100 people. One segment was selected at random by drawing lots. All the households in the selected segment were visited door-to-door and all eligible people (i.e. aged 7 years and older and residents at least 3 months) included until the sample size of 96 people was reached. If an eligible person was absent, the survey team revisited twice to interview the person before leaving the area.

### Data collection

Interviews and eye examinations were conducted in participants’ homes by trained interviewers in Kinyarwanda, which is the national language of Rwanda.

#### Questionnaire data

Heads of each household completed a questionnaire about household assets and housing characteristics (29 items) taken from the Rwanda living standards measurement survey.[20] In addition, we collected information on individual sociodemographic factors (education, literacy, and health insurance), self-rated health and self-rated vision for all participants. To ascertain the presence of eye symptoms indicative of rhinoconjunctivitis, we asked five questions extrapolated from the Rhinoconjunctivitis Quality of Life Questionnaire (RQLQ) on whether the participant had been troubled by itchy, watery, sore, swollen or sticky eyes in the past week, with answers on a 5 point scale (where 1 = not troubled ranging to 5 = extremely troubled).[21]

#### Eye examination

Presenting distance visual acuity (PVA) was assessed in both eyes of all participants using the smartphone based Portable Eye Examination Kit (PEEK). This method uses a tumbling “E” sequence and has been previously described and validated against gold standard.[22] All participants with visual acuity of less than 6/12 (logMAR 0.3) in the better eye underwent repeat visual acuity assessment using pinhole to assess the presence of refractive error. We assessed reading vision in all participants aged over 40 years using the Rwandan clinical near vision screening test, which uses a reading chart printed in the local language with N8 optotype and read at 40cm in ambient light. In line with local clinical practice, for those who were unable to complete the test as they could not identify words or numbers we used self-reported vision with answers on a 5-point scale, where we asked “Do you/does [name] have difficulty clearly seeing the picture on a coin, or threading a needle even if wearing glasses?”. Participants who reported having a lot of difficulty or cannot see it at all were categorized as needing reading glasses.

### Outcomes

The primary outcomes for this study are prevalence of distance URE and overall need for PEC. **Table 1** outlines the definitions of these outcomes, but broadly, need for PEC was defined as the presence of URE, need for reading glasses, VI requiring referral and/or other symptoms that could benefit from PEC (e.g. signs of conjunctivitis). Visual impairment was also measured, and defined as PVA worse than 6/12 in the better eye.[23] We also compared this more conservative cut off to the definition used by the WHO of PVA 6/18 or worse in the better eye.[24] Total presbyopia was measured and defined as:

- Participants needing reading glasses: cannot read N8 with good distance vision or poor self-rated vision (i.e. need for reading glasses/uncorrected presbyopia)
- Participants with glasses: can read N8 with good distance vision, or good self-rated vision (i.e. corrected presbyopia).

**Table 1:** Definitions of outcomes.

Outcome	Definition
URE	PVA worse than 6/12 (>logMAR 0.3) in the better eye that corrects to better than 6/12 (≤logMAR 0.3) with pinhole.
Need for reading glasses	• Aged over 40 and • PVA better than 6/12 (≤logMAR 0.3) in worse eye and • Unable to read N8 chart. Those who stated they could not read words or numbers and unable to read the chart when shown were also asked about self-rated vision. Those reporting cannot see at all, or great difficulty in seeing close objects were also categorized as need reading glasses.
VI requiring referral	PVA worse than 6/12 (>logMAR 0.3) in the better eye that does not correct to 6/12 with pinhole.
Symptoms that may benefit from PEC	Any symptom response of moderate or worse (graded 4 or more on the 5 point scale) to any individual questions in the symptoms questionnaire
Need for PEC	Using definitions above, participants fulfilling criteria for: • URE • Need for reading glasses • VI requiring referral • Symptoms that could benefit from PEC

URE = uncorrected refractive error; PVA = presenting visual acuity; PEC = primary eye care; LogMAR = logarithm of minimal angle of resolution; VI = visual impairment.

We measured spectacle coverage for both near and distance vision. For distance spectacle coverage we used the calculation: prevalence of refractive error- prevalence of URE/prevalence of refractive error. Prevalence of refractive error was estimated from both people with good presenting vision with distance glasses + people with URE. Presbyopia spectacle coverage was calculated as follows: prevalence of total presbyopia-prevalence of uncorrected presbyopia/total presbyopia. Presbyopia was estimated as people who could read N8 or good self reported near vision with reading glasses and people identified with a need for reading glasses.

### Training

Three teams of interviewers were trained for two weeks by an experienced trainer (DM) and an ophthalmologist (JY). Inter-observer agreement for VA assessment was measured through repeat examination of 60 patients by different team members to ensure PEEK measurement was comparable between team members (kappa≥0.60).

### Statistical analysis

To account for differential non-response according to age and sex, weighting was applied to the prevalence estimates to ensure they were representative of the population. Using Demographic Health Survey (DHS) estimates of the age and sex demographics of Rwanda, the expected population distribution was estimated by sex and 5-year age band. This was compared to the observed population distribution in the sample, and the expected probability of inclusion was divided by the observed probability of inclusion to give a weight for each age/sex category. These weights were then applied to the individuals who were observed in order to estimate the prevalence of each outcome. We generated a socioeconomic status (SES) score using principal component analysis of the household assets and housing characteristics, then classified into quartiles for a 4-point categorical variable. All outcomes (need for PEC, presence of URE) were binary variables. Logistic regressions were performed using each outcome as the dependent variable and using sex, education, SES, employment in the last month, and having health insurance as exposure variables. The univariable analysis was used to detect potential confounders, where statistically significant variables were included in a stepwise manner in the final weighted multivariable logistic regression model, and its significance in the final model tested using the Wald test. Age and sex were included as a priori confounders. All statistical analyses were conducted using Stata 14.

### Ethical approval and consent

This study was carried out according to the principles of the Declaration of Helsinki, with approval from the London School of Hygiene & Tropical Medicine ethics committee and the Rwandan National Ethics Committee. All participants gave written informed consent in Kinyarwanda or provided a thumbprint. All children aged 16 or under gave verbal assent and were interviewed in the presence of a caregiver who also provided written consent. All those we identified with potential to benefit from PEC in the survey, or with other healthcare needs, were referred to the local health center.

## Results

We enumerated 5,361 people in 50 clusters, of which 4618 participants were examined and interviewed (86% response rate). Of those who were enumerated but did not participate, 98% individuals were unavailable (not at home) and 2% refused. The majority of individuals who did not participate were males (61%). The age and sex distribution of the sample in comparison to that of the general population (from the census) is shown in **Table 2**. Compared to the Rwandan population, there was a higher proportion of females in our sample, a lower proportion of people aged 16–39 years, and a higher proportion of those aged 7–16 and 40+ years old. Therefore weights were applied to all subsequent analyses to provide estimates representative of the Rwandan population. Health insurance coverage in our sample (79.5%; 95%CI = 78.30, 80.65) was lower than recent estimates of health insurance coverage in Rwanda (91%).[25] The majority of people in our survey were covered by *Mutelles de Sante*, Rwanda’s community based health insurance (76.8%; 95%CI = 75.5, 78.0%) with a comparable proportion to that of the general population (81%).[25] Compared to the general Rwandan population, a higher proportion of our sample had completed primary school (14.1% vs 30.86% (95%CI = 29.54, 35.20)).[26]

**Table 2:** Characteristics of population survey participants (n = 4,618) (CI = confidence interval).

	N	% in sample (95%CI)	% in population
Age group (years)			
7–16	1,664	36.03 (34.66, 37.43)	28.60
17–24	778	16.85 (15.79, 17.95)	22.90
25–39	1,018	22.04 (20.87, 23.26)	26.40
40+	1,158	25.10 (23.85, 26.35)	22.20
Female	2,644	57.25 (55.82, 58.68)	51.80
Health insurance coverage	3,626	79.50 (78.30, 80.65)	90.00
Completion of primary school	1,425	30.86 (29.54, 35.20)	14.10

Complete data on visual acuity and all outcomes was collected on all participants. In our sample, 3.9% of participants were classed as visually impaired, providing a population prevalence estimate, after adjusting for age and sex, of 3.7% (95% CI: 3.0%, 4.5%). Using a more restrictive cut off of PVA 6/18 or worse, the prevalence was 1.6% (95%CI: 1.2, 2.2). The age and sex adjusted prevalence of VI and key outcomes in different age groups are shown in **Table 3.**

**Table 3:** Weighted and unweighted prevalence of URE, VI requiring referral, need for reading glasses, minor eye symptoms, visual impairment and total presbyopia.

	Weighted prevalence		Unweighted prevalence	
Outcome	%	95% CI	%	95% CI
URE (A)				
Overall	2.15	(1.65–2.81)	2.29	(1.90–2.77)
6–16 years	0.38	(0.17–0.86)	0.36	(0.16–0.80)
17–39 years	1.01	(0.60–1.70)	1.00	(0.63–1.59)
40+years	6.91	(5.39–8.80)	7.08	(5.74–8.71)
Total refractive error (corrected and uncorrected)	2.63	(2.08–3.33)		
Need for reading glasses (B) 40+ years	33.03	(29.38–36.90)	32.93	(30.28–35.70)
Presbyopia (corrected and uncorrected)	39.44	(35.76–43.24)	38.63	(35.87–41.48)
VI requiring referral (C)				
Overall	1.50	(1.16–1.94)	1.58	(1.26–1.98)
7–16 years	0.06	(0.01–0.44)	0.06	(0.01–0.43)
17–39 years	0.86	(0.51–1.43)	0.84	(0.50–1.38)
40+years	4.80	(3.61–6.34)	4.92	(3.81–6.33)
				
Symptoms (D)				
Overall	28.00	(25.78–30.33)	28.58	(27.30–29.91)
7–16 years	21.77	(19.02–24.77)	21.63	(19.72–23.68)
17–39 years	26.38	(23.81–29.13)	27.39	(25.38–29.51)
40+years	39.84	(35.87–43.94)	40.41	(37.62–43.27)
				
Need PEC (A-D)				
Overall	34.03	(31.78–36.35)	35.12	(33.76–36.51)
7–16 years	22.01	(19.25–25.05)	21.88	(19.95–23.93)
17–39 years	26.88	(24.30–29.62)	27.90	(25.87–30.02)
40+years	65.03	(60.82–69.02)	65.37	(62.58–68.02)
				
Mild Visual impairment (PVA 6/12 or worse)				
Overall	3.67	(2.98–4.49)	3.88	(3.36–4.47)
7–16 years	0.41	(0.21–0.93)	0.42	(0.20–0.88)
17–39 years	1.87	(1.28–2.74)	1.84	(1.31–2.57)
40+years	11.70	(9.64–14.13)	12.00	(10.25–14.01)
				
Moderate visual impairment (PVA 6/18 or worse)				
Overall	1.60	(1.19–2.15)	1.65	(1.32–2.06)
7–16 years	0.19	(0.06–0.60)	0.18	(0.06–0.56)
17–39 years	1.15	(0.67–1.95)	1.06	(0.68–1.65)
40+years	4.48	(3.30–6.04)	4.66	(3.59–6.04)
50 or over	6.37	(4.47–9.00)	6.74	(5.09–8.89)
Total presbyopia 40+ years)	44.77	(41.02–48.58)	44.68	(41.84–47.57)

CI = confidence interval PVA = presenting visual acuity VI = visual impairment URE = uncorrected refractive error.

### URE

The age-and sex-adjusted prevalence of URE was 2.2% (95% CI = 1.7, 2.8). Spectacle coverage for distance vision was 18.3% (95%CI = 13.8, 23.5). In univariable logistic regression, URE increased with age with 7% increase in odds of URE for each year (OR = 1.07; 95%CI = 1.06, 1.08). URE decreased with higher levels of education and SES (**Table 4**). URE prevalence increased with age. On multivariate analysis, there were no associations observed between URE and sex, education, having health insurance or SES (**Table 5)**.

**Table 4:** Weighted crude (univariable) associations with uncorrected refractive error (URE) and need for reading glasses.

		URE (n = 4618)			Need for reading glasses (n = 1158)		
		OR	95%CI	p-value	OR	95%CI	p-value
**Age**	**N**						
6–16 years	1664	Ref			Ref		
16–39 years	1796	2.66	(1.05, 5.78)	0.04			
40+ years	1158	19.27	(8.88, 41.83)	**<0.01**			
**Sex**							
Male	1974	Ref			Ref		
Female	2644	1.28	(0.76, 2.14)	0.35	1.27	(1.00, 1.62)	0.05
**Education**							
None/ preschool only	735	Ref			Ref		
Primary	3210	0.27	(0.18, 0.41)	**<0.01**	2.76	(1.79, 4.27)	**<0.01**
Post-primary or higher	673	0.15	(0.07		1.43	(0.85, 2.41)	0.17
**Urban or rural**							
Urban	634	Ref			Ref		
Rural	3984	1.78	(0.84, 3.76)	0.13	1.25	(0.66, 2.35)	0.49
**SES quartile** (27 missing values)							
1 (poorest)	1054	Ref			Ref		
2	1157	0.71	(0.41, 1.23)	0.22	1.06	(0.72, 1.55)	0.78
3	1189	0.86	(0.49, 1.49)	0.57	1.61	(1.08, 2.40)	0.02
4 (wealthiest)	1191	0.36	(0.18, 0.73)	**<0.01**	1.38	(0.87, 2.19)	0.17
**Health Insurance** (57 missing values)							
No	935	Ref			Ref		
Yes	3626	1.84	(0.93, 3.64)	0.08	0.87	(0.65, 1.18)	0.37
**Age (**Per year)	4168	1.07	(1.06, 1.08)	**<0.01**	1.00	(0.99, 1.01)	0.98

OR–Odds Ratio; 95%CI- 95% confidence intervals; SES- socioeconomic status; Ref = reference value; all p-values from Wald test, with significant values <0.01 highlighted in bold.

**Table 5:** Multivariable analysis of key outcomes in 4591 people aged 7–100 (URE, referrals, symptoms and need for PEC) and 1152 people aged 40–100 (need for reading glasses), adjusting for age, sex, education and socioeconomic status.

	URE			Need for reading glasses			Referrals					Symptoms				Need for PEC				
	OR	95%CI	p-value	OR	95%CI	p-value	OR	95%CI			p-value	OR	95%CI		p-value	OR		95%CI		p-value
**Age (per year)**	1.06	91.05, 1.08)	**<0.01**	1.01	(1.00, 1.02)	0.18	1.07	(1.05, 1.09)			**<0.01**	1.02	(1.01, 1.02)		**<0.01**	1.04		(1.04, 1.05)		**<0.01**
**Sex**																				
Male	Ref			Ref			Ref					Ref				Ref				
Female	1.08	(0.63, 1.85)	0.77	1.50	(1.18, 1.92)	**<0.01**	1.80	(1.02, 3.18)			0.04	1.22	(1.07, 1.39)		**<0.01**	1.21		(1.06, 1.38)		**<0.01**
**Education**																				
None/ only preschool	Ref			Ref			Ref					Ref				Ref				
Primary	1.02	(0.61, 1.72)	0.93	3.15	(1.96, 5.04)	**<0.01**	0.72	(0.37, 1.39)			0.32	0.85	(0.70, 1.03)		0.10	1.13		(0.90, 1.41)		0.29
Post-primary or higher	0.89	(0.35, 2.23)	0.80	1.66	(0.89, 3.10)	0.11	1.04	(0.33, 3.30)			0.94	0.86	(0.66, 1.13)		0.27	0.99		(0.77, 1.29)		0.96
**SES quartile** (27 missing values)																				
1 (poorest)	Ref			Ref			Ref					Ref				Ref				
2	0.81	(0.47, 1.41)	0.45	0.96	(0.65, 1.42)	0.84	1.45	(0.73, 2.91)			0.28	1.02	(0.80, 1.29)		0.89	1.01		(0.79, 1.29)		0.94
3	0.95	(0.56, 1.60)	0.83	1.48	(0.97, 2.26)	0.07	0.79	(0.36, 1.75)			0.56	0.93	(0.72, 1.21)		0.60	1.05		(0.83, 1.34)		0.67
4 (wealthiest)	0.57	(0.27, 1.20)	0.13	1.15	(0.70, 1.89)	0.57	0.76	(0.30, 1.88)			0.54	1.05	(0.78, 1.41)		0.72	1.04		(0.77, 1.40)		0.78

URE = uncorrected refractive error; PEC = primary eye care; OR = odds ratio; CI = confidence interval; SES = socioeconomic status.

### Need for reading glasses

Of the 1158 participants who were aged 40 and over, 351 participants could not read nor recognize numbers, and 807 (69.7%) completed the test. Of the 351 participants who could not complete the test, 33 (9.4%) reported a lot of difficulty and five (1.4%) could not see detailed objects at reading distance. We categorized those who could not read N8 or had poor self-reported near vision, but with good distance vision (can see 6/12) as needing reading glasses.

Based on both these tests, we found over a third of the population aged 40 and over would benefit from reading glasses (33.0%, 95%CI = 29.4, 36.9). The population prevalence of presbyopia in people aged 40 and over was 39.4% (95%CI = 35.8, 43.2%) based on those we identified with need for reading glasses and participants with glasses who were able to read or had good reported near vision, with good distance vision. The presbyopic spectacle coverage was 16.3% (95%CI = 15.1, 17.4).

In univariable logistic regression, need for reading glasses was 34% higher in females compared to males (OR = 1.27; 95%CI = 1.00, 1.62) and more than twice as high in those with primary level vs no education (OR = 2.76; 95%CI = 1.79, 3.11) (**Table 4**). In multivariable analysis females and higher levels of education were associated with need for presbyopic correction (OR = 1.50, 95%CI = 1.18–1.92 for females; OR = 3.15, 95%CI = 0.89, 3.10 for primary level education compared to none) **(Table 5**).

### Need for referrals to secondary care

Population prevalence of need for referral from PEC to secondary care was 1.5% (95%CI = 1.2, 1.9%) consisting of people who had PVA<6/12 in better eye, which could not be corrected with pinhole. The need for referrals increased with age with less than 1% in those under 40 and rising to 4.8% (3.6, 6.3%) in those aged 40 and above who would require referrals from PEC.

On univariate logistic regression, women, older people, and people with lower levels of educational attainment were more likely to require referral (**Table 6**). However, only age and female gender were independently associated with referrals in a weighted multivariable regression analysis, with females have 80% increased odds of requiring referrals compared to men (OR = 1.80, 95%CI = 1.02–3.18) (**Table 5**).

**Table 6:** Weighted crude (univariable) associations with referrals, symptoms and need for primary eye care.

	Referrals (n = 4618)			Symptoms (n = 4618)			Need for Primary Eye Care (n = 4618)		
	OR	95% CI	p-value	OR	95% CI	p-value	OR	95% CI	p-value
									
**Age**									
6–16 years	Ref			Ref			Ref		
16–39 years	14.97	(1.80, 124,49)	**0.01**	1.29	(1.11, 1.50)	**<0.01**	1.30	(1.12, 1.52)	**<0.01**
40+ years	86.90	(10.96,689.30)	**<0.01**	2.38	(1.94, 2.92)	**<0.01**	6.59	(5.29, 8.20)	**<0.01**
**Sex**									
Male	Ref			Ref			Ref		
Female	2.15	(1.28, 3.62)	**<0.01**	1.30	(1.14, 1.48)	**<0.01**	1.30	(1.15, 1.47)	**<0.01**
**Education**									
None/ only preschool	Ref			Ref					
Primary	0.19	(0.11, 0.30)	**<0.01**	0.61	(0.51, 0.73)	**<0.01**	0.54	(0.44, 0.65)	**<0.01**
Post-primary or higher	0.16	(0.06, 0.40)	**<0.01**	0.63	(0.49, 0.81)	**<0.01**	0.48	(0.38, 0.60)	**<0.01**
**Urban or rural**									
Urban	Ref			Ref			Ref		
Rural	1.33	(0.64, 2.74)	0.44	0.87	(0.66, 1.15)	0.33	1.00	(0.75, 1.33)	0.99
**SES quartile** (27 missing values)									
1 (poorest)	Ref						Ref		
2	1.10	(0.58, 2.09)	0.76	0.98	(0.78, 1.24)	0.87	0.98	(0.79, 1.21)	0.82
3	0.69	(0.33, 1.44)	0.32	0.90	(0.70, 1.15)	0.39	1.01	(0.82, 1.25)	0.91
4 (wealthiest)	0.42	(0.18, 1.01)	0.05	0.95	(0.72, 1.25)	0.70	0.88	(0.68, 1.13)	0.31
**Health Insurance** (57 missing values)									
No	Ref						Ref		
Yes	0.84	(0.51, 1.390	0.49	0.89	(0,74, 1.06)	0.19	0.95	(0.80, 1.14)	0.59
Age (per year)	1.07	(1.06, 1.08)	<0.01	1.02	(1.01, 1.02)	**<0.01**	1.04	(1.04, 1.05)	**<0.01**

OR–Odds Ratio; 95%CI- 95% confidence intervals; SES- socioeconomic status; Ref = reference value; all p-values from Wald test, with significant values <0.01 highlighted in bold.

### Symptoms

Over a quarter of the participants reported symptoms that could benefit from PEC (28.0%; 95%CI = 25.8–30.3%), with higher levels of need in those who were older (Table 3). The most common symptom was itch (21.8%; 95%CI = 20.6, 22.9) and watery eyes (17.4%; 95%CI = 16.3, 18.5) indicative of allergy related conjunctivitis, with lower levels of need reported for sore (1.2%; 95%CI = 9.2, 15.5) swollen (3.3%; 95%CI = 2.8, 3.9) or sticky eyes (5.9%; 95%CI = 5.3, 6.6), which are suggestive of infectious causes (**Table 7**).

**Table 7:** Proportion of participants presenting with conjunctivitis symptoms. (95%CI -95% confidence interval).

(N = 4618)	n	%	95% CI
Itch	1005	21.8	20.6, 22.9
Watery	803	17.4	16.3, 18.5
Sore	55	1.2	9.2, 15.5
Swollen	153	3.3	2.8, 3.9
Sticky	273	5.9	5.3, 6.6

Females were 30% more likely to report moderate or worse symptoms than males (OR = 1.30; 95%CI = 1.14, 1.48) (Table 5). An increase in self-reported symptoms was also seen with age (OR = 1.02; 95%CI = 1.01, 1.02) (**Table 6**). No further associations were found. Those with higher levels of educational attainment had lower odds of reporting symptoms requiring PEC (OR = 0.61 95%CI = 0.51, 0.73 primary vs none and OR = 0.63; 95%CI = 0.49, 0.81 post primary vs none), though this was not statistically significant in multivariable analysis (**Table 5**).

### Need for PEC

Overall, the estimated need for PEC in the population was 34.0% (95%CI = 31.7, 36.3%) (**Table 3**). The need was greatest in those over 40 (65.0%; 95%CI = 60.8, 69.0), and was higher in females than males (OR = 1.30 (95%CI = 1.15, 1.47) (**Table 6**). Need was lower in those with higher levels of education, however this association did not remain on multivariate analysis (**Table 5**). There was no evidence of an association between need for PEC and SES or health insurance status.

## Discussion

Universal access to primary care and integrated primary eye care is a key global initiative to reduce preventable causes of blindness.[27] While there have been large numbers of VI surveys in those aged 50, there is little information for younger age groups or on the proportion of a population who could benefit from primary eye care. In this national survey of background need for primary eye care, we found a high need for PEC with over a third (34.0%; 95%CI = 30.5–35.0%) of the population with symptoms or signs of eye health problems that had the potential to benefit from PEC offered at the local health centre. In contrast, the overall adjusted prevalence of VI (3.7%, 95%CI = 3.0, 4.5) and URE was low (2.4%, 95%CI = 1.9, 3.1) in this population aged 7 years and above. Coverage of spectacles was low for both presbyopia (19.1%) and distance vision (18.1%).

Two previous surveys of blindness have been conducted in Rwanda, to allow comparison of results. A Rapid Assessment of Avoidable Blindness (RAAB) survey conducted in the Western province in 2006 estimated VI (VA worse than 6/18) prevalence of 8.4% (not including presbyopia) amongst those over 50 years old, in comparison to our estimated 6.4% (4.5–9.0%).[28] A more recent RAAB from 2015 detected VI prevalence of 4.9%, which is lower than our estimates.[29]

The high level of need for PEC due to symptoms confirms previous work from Nigeria which found that 25.2% of the population had ocular morbidity in at least one eye, and the leading causes were presbyopia and conditions affecting the lens and conjunctiva.[11] The most common cause of need for PEC in our study was symptoms consistent with conjunctivitis, with allergy symptoms such as moderate or severe itch in nearly a quarter of the population (21.8%). Previous studies in the sub-Saharan African region have also found allergic conjunctivitis is common in school-aged children. A high prevalence of allergic conjunctivitis was observed in a study of school children in urban Ghana (39.9%) and the more serious form, vernal keratoconjunctivitis was detected in 4.0% of school children in Rwanda.[30] [31] Allergic conjunctivitis can be treated simply with eyedrops, with more serious cases requiring referrals to an ophthalmologist. This common condition can be treated in PEC and would improve access and quality of life for these children experiencing symptoms.

URE can also be detected in PEC. Resnikoff et al. (2008) conducted a systematic review of population studies to extrapolate and estimate the magnitude of visual impairment from URE.[32] In the review, the prevalence of VI (using 6/18 as a cut-off) from URE in Africa was 1% in those aged 5 and above, which is comparable to our results presented in this paper (1.6%).[32] Spectacle coverage for those with distance URE was low in our study (18.1%). Compared to other countries in sub-Saharan African where estimates have been made, our estimate was higher than The Gambia (7.1%) and Guinea Bissau (14.8%), and Eritrea (17.6%), however lower than coverage in Botswana (53.7%).[8] Despite the positive results in comparison to other countries of the region, coverage is still low and thus further efforts to scale up URE correction in PEC is required in Rwanda.

A third of people aged 40 years and older in this survey had a need for reading glasses, with need increasing with age, as expected and observed in other studies.[5, 33–35] Higher educational attainment was also associated with need for reading glasses, and confirms findings from Tanzania and Eritrea.[33, 34] We used the current Rwandan near vision chart utilized in PEC, and completing this test required literacy, supplemented with a self-reported near vision for those who could not read. This may have underestimated the potential to benefit from near correction in this population. However, our population estimates for presbyopia (57.4%) were comparable to the prevalence of presbyopia found in studies in Tanzania (61.9%), but differed from that in Eritrea and Kenya at 32.9%, and 85.4% respectively. [5, 33–35] We found a higher presbyopic spectacle coverage of 13.9% compared to other studies from Africa of 0.5% and 4.8% in Tanzania and Durban, South Africa respectively (18,19), but lower than that observed in over 50 year olds in Zanzibar (17.6%).[36] This suggests that the current provision of presbyopic correction through PEC has reached a higher proportion of the population compared to some African countries, though coverage is low and there is a continued need to scale up presbyopia correction services.

This is the first study to measure the all-age prevalence of URE and need for PEC in Rwanda and offers valuable insights to public health planning. There are 16 ophthalmologists and 65 ophthalmic technicians in Rwanda, covering a predominantly rural population of 11.5 million people. Since 2012, general nurses at local health centres have been trained to provide PEC, with the aim of improving access to eye health services. The results from this study show that conditions that can be treated in PEC, such as presbyopia and conjunctivitis, form an important healthcare need in the population. Therefore, the provision of PEC has the potential to reduce unnecessary demand for specialized services and allow a more efficient use of scarce resources.[37]

There are some limitations in this study. The proportions of children and women differed in our study sample compared to that of the Rwandan population. In addition, a higher proportion of non-responders in our study were male, likely due to participation in income generating activities. We adjusted for age and sex in our final prevalence estimates, which would mitigate the effect of these potential biases. Level of education of the participants in our sample was higher than the general population of Rwanda. Given that higher levels of education was associated with lower chance of reporting symptoms and URE, the prevalence of these outcomes may be underestimated in the survey. Higher education was also associated with increased need for reading glasses, potentially overestimating this component of need. We used visual acuity cut offs aligned to that used in PEC to detect those who had the potential to benefit, therefore the use of 6/12 (LogMAR 0.3) is a lower threshold and will identify more people with VI compared to WHO definitions of VI. However, global visual impairment estimates have recently shifted towards using 6/12 as a cut off.[23] Our use of a validated mobile application to assess VA facilitated our pragmatic approach. We did not apply cycloplegia or retinoscopy to younger participants, and this is likely to result in an underestimate of URE. However, these methods detect lower levels of refractive error and we were unlikely to miss people with visual impairment due to URE in our study. Participants were not examined clinically for the presence of conjunctivitis or other ocular symptoms for practical reasons. However, we used a validated questionnaire to determine self-reported symptoms to ensure accuracy of results.

## Conclusions

Scarce resources require careful planning. Our findings support the need to provide access to PEC. Nearly a third of the population in Rwanda could benefit from PEC, mainly driven by ocular symptoms and the need for reading glasses in those older than 40 years. Women were more likely to need PEC and require a referral, indicating more serious eye conditions, such as cataract. As women are more likely to be caregivers and stay close to home, when and where PEC is provided must be considered in planning to ensure equity of access. Despite progress in provision of PEC in Rwanda, the next step is to examine how well people access PEC, and the impact of PEC on eye health outcomes.

## Supporting information

S1 Table Crude (univariable) associations with uncorrected refractive error (URE) and need for reading glasses. (DOCX)

S2 Table Crude (univariable) associations with referrals, symptoms and need for primary eye care. (DOCX)
