# Supplementary material for: Population need for primary eye care in Rwanda: A national survey
Source: PLoS One. 2018 May 1;13(5):e0193817. doi: 10.1371/journal.pone.0193817 (PMC5929506; doi:10.1371/journal.pone.0193817)
Supplement: S1 Table — (DOCX) [file pone.0193817.s001.docx]

**S1 Table.** Crude (univariable) associations with uncorrected refractive error (URE) and need for reading glasses.

|  |  | **URE** | | | **Need for reading glasses** | | |
| --- | --- | --- | --- | --- | --- | --- | --- |
|  |  | **OR** | **95%CI** | **p-value** | **OR** | **95%CI** | **p-value** |
| **Age** | **N** |  |  |  |  |  |  |
| 6-16 years | 1664 | Ref |  |  | Ref |  |  |
| 16-39 years | 1796 | 2.80 | (1.11, 7.06) | 0.03 |  |  |  |
| 40+ years | 1158 | 21.06 | (9.16,48.41) | **<0.01** |  |  |  |
| **Sex** |  |  |  |  |  |  |  |
| Male | 1974 | Ref |  |  | Ref |  |  |
| Female | 2644 | 1.34 | (0.90, 2.01) | 0.15 | 1.34 | (1.05, 1.72) | 0.02 |
| **Education** |  |  |  | **<0.01** |  |  |  |
| None/ preschool only | 735 | Ref |  |  | Ref |  |  |
| Primary | 3210 | 0.25 | (0.17, 0.38) | **<0.01** | 2.39 | (1.84, 3.11) | **<0.01** |
| Post-primary or higher | 673 | 0.16 | (0.07, 0.35) | **<0.01** | 1.07 | (0.69, 1.66) | 0.76 |
| **Urban or rural** |  |  |  |  |  |  |  |
| Urban | 634 | Ref |  |  | Ref |  |  |
| Rural | 3984 | 1.73 | (0.87, 3.45) | 0.12 | 1.29 | (0.85, 1.96) | 0.24 |
| **SES quartile**  (27 missing values) |  |  |  |  |  |  |  |
| 1 (poorest) | 1054 | Ref |  |  | Ref |  |  |
| 2 | 1157 | 0.64 | (0.38, 1.08) | 0.10 | 0.97 | (0.70, 1.35) | 0.87 |
| 3 | 1189 | 0.81 | (0.49, 1.31) | 0.38 | 1.52 | (1.10, 2.09) | 0.01 |
| 4 (wealthiest) | 1191 | 0.35 | (0.19, 0.65) | **<0.01** | 1.19 | (0.83, 1.70) | 0.35 |
| **Health Insurance**  (57 missing values) |  |  |  |  |  |  |  |
| No | 935 | Ref |  |  | Ref |  |  |
| Yes | 3626 | 1.83 | (1.02, 3.28) | 0.05 | 0.87 | (0.64, 1.18) | 0.36 |
| **Age**  Per year | 4168 | 1.07 | (1.06, 1.08) | **<0.01** | 1.02 | (1.01, 1.03) | **<0.01** |

OR – Odds Ratio; 95%CI- 95% confidence intervals; SES- socioeconomic status; Ref=reference value; all p-values from Wald test, with significant values <0.01 highlighted in bold.
