# Supplementary material for: Population need for primary eye care in Rwanda: A national survey
Source: PLoS One. 2018 May 1;13(5):e0193817. doi: 10.1371/journal.pone.0193817 (PMC5929506; doi:10.1371/journal.pone.0193817)
Supplement: S2 Table — (DOCX) [file pone.0193817.s002.docx]

**S2 Table.** Crude (univariable) associations with Referrals, Symptoms and Need for Primary Eye Care.

|  | **Referrals** | | | **Symptoms** | | | **Need for Primary Eye Care** | | |
| --- | --- | --- | --- | --- | --- | --- | --- | --- | --- |
|  | **OR** | **95% CI** | **p-value** | **OR** | **95% CI** | **p-value** | **OR** | **95% CI** | **p-value** |
|  |  |  |  |  |  |  |  |  |  |
| **Age** |  |  |  |  |  |  |  |  |  |
| 6-16 years | Ref |  |  | Ref |  |  | Ref |  |  |
| 16-39 years | 14.01 | (1.85, 106.15) | **0.01** | 1.37 | (1.17, 1.60) | **<0.01** | 1.38 | (1.18, 1.61) | **<0.01** |
| 40+ years | 86.10 | (11.9, 622.66) | **<0.01** | 2.46 | (2.08, 2.90) | **<0.01** | 6.74 | (5.70, 7.97) | **<0.01** |
| **Sex** |  |  |  |  |  |  |  |  |  |
| Male | Ref |  |  | Ref |  |  | Ref |  |  |
| Female | 2.15 | (1.27, 3.63) | **<0.01** | 1.27 | (1.11, 1.44) | **<0.01** | 1.28 | (1.13, 1.44) | **<0.01** |
| **Education** |  |  |  |  |  |  |  |  |  |
| None/ preschool only | Ref |  |  | Ref |  |  | Ref |  |  |
| Primary | 0.18 | (0.11, 0.29) | **<0.01** | 0.57 | (0.49, 0.68) | **<0.01** | 0.50 | (0.42, 0.59) | **<0.01** |
| Post-primary or higher | 0.17 | (0.07, 0.40) | **<0.01** | 0.62 | (0.49, 0.77) | **<0.01** | 0.48 | (0.38, 0.59) | **<0.01** |
| **Urban or rural** |  |  |  |  |  |  |  |  |  |
| Urban | Ref |  |  | Ref |  |  | Ref |  |  |
| Rural | 1.30 | (0.62, 2.72) | 0.49 | 0.86 | (0.71, 1.03) | 0.09 | 0.99 | (0.83, 1.18) | 0.91 |
| **SES quartile**  (27 missing values) |  |  |  |  |  |  |  |  |  |
| 1 (poorest) | Ref |  |  | Ref |  |  | Ref |  |  |
| 2 | 1.04 | (0.58, 1.88) | 0.89 | 0.95 | (0.79, 1.14) | 0.56 | 0.95 | (0.80, 1.13) | 0.55 |
| 3 | 0.71 | (0.37, 1.36) | 0.31 | 0.92 | (0.77, 1.11) | 0.38 | 1.05 | (0.88, 1.24) | 0.61 |
| 4 (wealthiest) | 0.42 | (0.20, 0.89) | 0.02 | 0.94 | (0.79, 1.13) | 0.52 | 0.87 | (0.73, 1.04) | 0.13 |
| **Health Insurance**  (57 missing values) |  |  |  |  |  |  |  |  |  |
| No | Ref |  |  | Ref |  |  | Ref |  |  |
| Yes | 0.90 | (0.51, 1.58) | 0.72 | 0.91 | (0.78, 1.07) | 0.25 | 0.98 | (0.84, 1.14) | 0.79 |
| Age (per year) | 1.07 | (1.06, 1.09) | <**0.01** | 1.02 | (1.02, 1.02) | **<0.01** | 1.04 | (1.04, 1.05) | **<0.01** |

OR – Odds Ratio; 95%CI- 95% confidence intervals; SES- socioeconomic status; Ref=reference value; all p-values from Wald test, with significant values <0.01 highlighted in bold.
